# Neutrophil–Lymphocyte Ratio and KELIM Score as Prognostic Markers in High-Grade Serous Advanced Ovarian Cancer Patients Treated with Neoadjuvant Chemotherapy

**DOI:** 10.3390/biomedicines13040975

**Published:** 2025-04-16

**Authors:** Vasilis Theodoulidis, Kalliopi Kissoudi, Kimon Chatzistamatiou, Panagiotis Tzitzis, Dimitris Zouzoulas, Iakovos Theodoulidis, Christos Anthoulakis, Theodoros Moysiadis, Maria Topalidou, Eleni Timotheadou, Grigoris Grimpizis, Dimitris Tsolakidis

**Affiliations:** 1 1st Department of Obstetrics & Gynecology, Aristotle University of Thessaloniki, General Hospital of Thessaloniki “Papageorgiou” Greece, 56429 Thessaloniki, Greece; kissoudi.c@gmail.com (K.K.); kimon.chatzistamatiou@gmail.com (K.C.); ptzitzis@gmail.com (P.T.); dzouzoulas@hotmail.gr (D.Z.); iakwbostheo@gmail.com (I.T.); christos.anthoulakis@hotmail.com (C.A.); grigoris.grimbizis@gmail.com (G.G.); dtgyn@auth.gr (D.T.); 2 Department of Computer Science, School of Sciences and Engineering, University of Nicosia, 2417 Nicosia, Cyprus; moysiadis.t@unic.ac.cy; 3 Radiotherapy Department, “Papageorgiou” Hospital, 56429 Thessaloniki, Greece; mariatop@gmail.com; 4 Department of Oncology, Aristotle University of Thessaloniki, Genaral Hospital of Thessaloniki “Papageorgiou” Greece, 56429 Thessaloniki, Greece; timotheadou@auth.gr

**Keywords:** advanced ovarian cancer, neoadjuvant chemotherapy, neutrophil–lymphocyte ratio, CA-125 KELIM, survival outcomes, recurrence

## Abstract

**Background/Objectives**: Advanced ovarian cancer (AOC) is frequently diagnosed at late stages, with a 5-year overall survival (OS) rate of approximately 25%. While primary debulking surgery followed by chemotherapy remains the standard treatment, neoadjuvant chemotherapy (NACT) followed by interval debulking surgery (IDS) is an alternative for patients with extensive disease. Achieving complete cytoreduction is a critical prognostic factor for OS and progression-free survival (PFS). This study evaluated the prognostic value of two biomarkers—the neutrophil–lymphocyte ratio (NLR) and the cancer antigen-125 (CA-125) ELIMination rate constant K (KELIM)—in predicting survival outcomes and recurrence rates in patients with AOC undergoing NACT. **Methods**: A retrospective, single-center analysis was conducted on 78 patients with high-grade serous AOC (stages III–IV) treated with platinum-based NACT followed by IDS between January 2013 and December 2023. NLR was calculated from prechemotherapy complete blood counts, with a threshold of ≥3 indicating elevated levels. KELIM, a marker of tumor chemosensitivity, was derived from CA-125 kinetics during the first 100 days of chemotherapy, with a cutoff of ≥1 denoting a favorable outcome. Clinical outcomes, including PFS and OS were analyzed using Kaplan–Meier survival curves, log-rank tests, and Cox regression models. **Results**: Results demonstrated that elevated NLR (≥3) and low KELIM (<1) were associated with poorer PFS and OS. KELIM score was identified as a strong prognostic marker for both PFS and OS, while NLR demonstrated weak association. Complete cytoreduction was achieved in 69.2% of patients, significantly correlating with improved survival outcomes. Postoperative complications, assessed using the Clavien–Dindo classification, were observed in a small subset of patients, with a total median hospital stay of 8 days. **Conclusions**: This study highlights the potential of NLR and KELIM as prognostic tools in AOC, aiding in patient selection for radical surgical interventions and predicting chemosensitivity. Future multicenter studies with larger cohorts are needed to validate these results and further explore the clinical utility of these biomarkers in optimizing treatment strategies for AOC.

## 1. Introduction

Ovarian cancer is often diagnosed at an advanced stage (III and IV), with a 5-year overall survival rate of approximately 25% [1]. The standard treatment for advanced ovarian cancer (AOC) involves primary surgical cytoreduction followed by platinum and taxane-based chemotherapy [2]. An alternative approach is neoadjuvant chemotherapy (NACT) followed by interval surgical cytoreduction, particularly in cases where complete cytoreduction is not feasible due to an extensive cancer burden [3]. Numerous studies have assessed these two strategies, comparing their efficacy, safety profiles, and survival outcomes [4,5]. The noninferiority trials of primary surgical cytoreduction versus NACT showed that tumor debulking to R0 was the most important indicator of overall survival (OS), and rates were higher in the NACT-interval surgical cytoreduction treatment arms [4,5,6].

Therefore, the use of predictive models to assess surgical outcomes and prognosis is essential for optimizing patient selection and identifying those who are most likely to benefit from extensive surgical interventions. Among the potential prognostic factors being explored are the cancer antigen-125 (CA-125) ELIMination rate constant K (KELIM) and the neutrophil–lymphocyte ratio (NLR).

The CA-125 KELIM (a kinetic parameter derived from CA-125 measurements within the initial 100 days of systemic chemotherapy) has been identified as a predictor of tumor intrinsic chemosensitivity [7]. KELIM represents the rate of CA-125 decline during chemotherapy, with a higher KELIM score indicating greater chemosensitivity. It has been identified as a biomarker for survival outcomes, including progression-free survival (PFS) and OS [7,8,9,10]. Additionally, KELIM has been associated with the likelihood of complete resection at interval debulking surgery and the risk of subsequent platinum-resistant relapse [11,12].

Inflammation has been identified as a critical factor in the initiation and progression of various solid tumors [13,14]. A range of inflammatory serum markers has been studied to evaluate their association with clinical outcomes and prognosis across different cancer types [15]. Among these, NLR has emerged as a potential marker for survival outcomes in ovarian cancer and other solid malignancies [16,17]. It is calculated as neutrophil count divided by lymphocyte count and can be easily derived from a complete blood count (CBC). Elevated NLR values at the time of diagnosis are associated with poorer PFS and OS [18,19], greater disease severity, and resistance to platinum-based therapy [20,21].

The aim of the present study was to evaluate the prognostic value of the KELIM score and the NLR values in predicting survival outcomes, including OS and PFS in women with high-grade serous AOC receiving NACT. While other markers such as the platelet–lymphocyte ratio (PLR) or BRCA mutation status hold prognostic value, our study aimed to address a gap in the literature by directly comparing NLR with KELIM, as no prior studies had evaluated their roles. This novel comparative approach was implemented to generate new insights into their individual and potentially complementary predictive capabilities

Our findings indicated that the KELIM score emerged as a strong prognostic marker for both PFS and OS, while NLR exhibited a weak association. Notably, the assessment of these two markers enhances prognostic accuracy, suggesting their potential complementary role in clinical practice. To the best of our knowledge, no prior study has directly compared these prognostic factors in this specific context, underscoring the novelty and clinical significance of our findings.

## 2. Materials and Methods

A retrospective, single-center cohort study was conducted at a tertiary institution to examine patients with stage III or IV ovarian or fallopian tube cancer who received platinum-based NACT followed by IDS over an 11-year period (January 2013–December 2023). All surgeries were performed by two specialized gynecologic oncologists, adhering to the guidelines of the European Society of Gynecological Oncology (ESGO) and prioritizing maximal efforts to achieve no residual disease. Ethical approval for the study was obtained from the hospital’s health ethics committee.

The inclusion criteria specified patients with newly diagnosed advanced ovarian cancer of high-grade serous histology who had received 3 to 4 cycles of neoadjuvant chemotherapy prior to interval debulking surgery, based on individualized clinical decisions such as performance status, tumor burden, and response to initial treatment. Patients who (i) underwent primary debulking surgery (PDS), (ii) had recurrent ovarian cancer, (iii) had incomplete registry data, or (iv) lacked follow-up attendance were excluded from the study.

During the study period, 324 patients were identified. Of these, 196 were excluded because they had undergone primary debulking surgery or had presented with recurrent ovarian cancer. An additional 50 patients were excluded due to missing essential registry data or discontinuation of follow-up. Ultimately, 78 patients with high-grade serous advanced ovarian cancer met the eligibility criteria and were included in the final analysis.

All data were collected within one month from the hospital’s computerized patient records system. Patients′ demographic and clinical characteristics included age, body mass index (BMI), Charlson comorbidity index (CCI) [22], serial CA-125 values during neoadjuvant chemotherapy, KELIM score, intensive care unit (ICU) admission, Clavien–Dindo classification for postoperative complications [23], length of hospital stay, residual disease status after debulking surgery based on the peritoneal cancer index (PCI) score, date of diagnosis, date of recurrence or disease progression, and date of last follow-up or death.

The KELIM score was calculated in the neoadjuvant setting using an available online tool [24]. It was evaluated both as a continuous variable and as a binary index, with a cut-off of ≥1 indicating a favorable outcome. The dates of each chemotherapy cycle, along with the corresponding CA-125 values, recorded within the first 100 days from the initiation of neoadjuvant chemotherapy, were input for analysis. Preferably, CA-125 values obtained before the 2nd, 3rd, and 4th chemotherapy cycles were used for the calculation. However, when these values were unavailable, the CA-125 measurement taken prior to the first chemotherapy cycle (within 7 days of starting neoadjuvant chemotherapy) was used. This adjustment was necessary for only seven patients.

NLR was determined using CBC data obtained from all patients before the initiation of chemotherapy. NLR was calculated by dividing the absolute neutrophil count by the absolute lymphocyte count. Consistent with prior research, an NLR greater or equal to 3 was considered elevated and associated with unfavorable outcomes [18,20,25,26,27,28].

Standard descriptive statistics were performed for both quantitative (mean and standard deviation) and qualitative variables (frequency and percentage). Kaplan–Meier curves were developed for PFS and OS according to the KELIM score and NLR values, using the “survfit” R function with the “survival” R package and the “ggsurvplot” R function with the “survminer” R package. The log-rank test was used to compare the corresponding survival distributions. For the KELIM score, the threshold of one was applied, and it was assessed as a binary variable (0: <1, 1: ≥1). For NLR, the threshold of three was used, and it was similarly assessed as a binary variable (0: <3, 1: ≥3). In the case of NLR, receiver operating characteristic (ROC) analysis and maximally selected rank statistics employing the “maxstat.test” function with the R package “maxstat” were additionally used in order to evaluate whether the threshold of three was appropriate based on our dataset.

Univariable Cox regression analysis was performed to assess the risk of recurrence (PFS) and OS according to the KELIM score and NLR values. Univariable Cox regression was used as well to evaluate the impact of age, BMI, CCI, ICU admission, Clavien–Dindo classification, and hospital stay. Variables exhibiting a statistically significant association with the outcome (*p*-value ≤ 0.05) would have then been included in a multivariable Cox regression model along with KELIM or NLR to account for potential confounders. The level of statistical significance was set at 0.05 for all analyses. For the descriptive statistics, the ROC and Cox regression analyses were performed using SPSS V22. All the remaining analyses were conducted with R V4.2.2, as indicated above.

## 3. Results

A total of 78 patients with high-grade serous advanced ovarian cancer were included. The mean age at diagnosis was 61.45 ± 12.32 years, and the mean BMI was 28.25 ± 5.92 kg/m^2^ (Table 1). The performance status of the patients, assessed using the CCI, revealed that 38 out of 78 patients (48.7%) had a CCI score of ≥3, indicating that nearly half of the cohort had mild to moderate comorbidities (Table 1). Among the included patients, 63 were diagnosed with stage III disease, and 15 had stage IV disease. Regarding treatment, 70 patients received three cycles of platinum-based chemotherapy, while 8 received four cycles.

Residual disease was assessed using the PCI, calculated both at the start and end of cytoreductive surgery. Fifty-four patients (69.2%) achieved complete cytoreduction with no residual disease, fourteen patients (17.9%) had residual disease measuring <1 cm, and ten patients (12.8%) underwent suboptimal debulking surgery with residual disease ≥1 cm.

Postoperative complications were evaluated using the Clavien–Dindo classification system, with a median score of 24.20 and an interquartile range (IQR) of 12.20–32.00. Fourteen patients required ICU admission, and the median hospital stay was 8 days, with an IQR of 6–9 days (Table 1).

**Figure 1:**
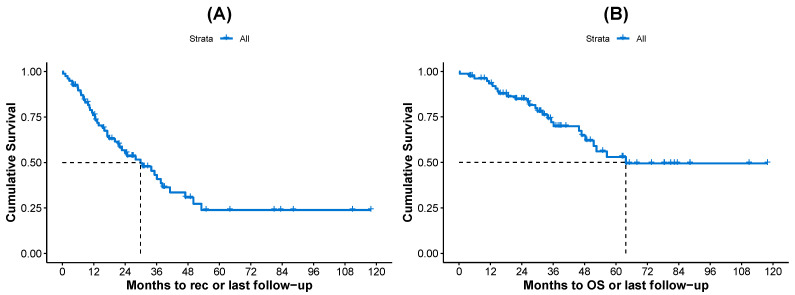
Kaplan–Meier curves for all patients, (**A**) PFS and (**B**) OS. The median survival times (dashed lines) are also displayed in each plot.

The median PFS and OS times were found to be 29.86 and 63.74 months, respectively, in the whole cohort (Table 1). The corresponding Kaplan–Meier curves are shown in Figure 1.

**Figure 2:**
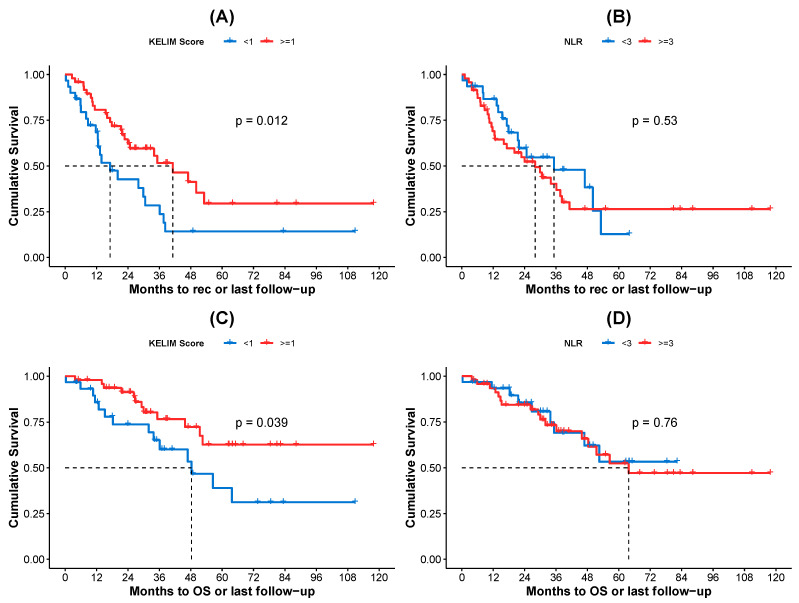
Kaplan–Meier curves for (**A**) PFS for patients according to KELIM score, (**B**) PFS for patients according to NLR values, (**C**) OS for patients according to KELIM score, and (**D**) OS for patients according to NLR values. Τhe KELIM score was treated as binary variable with threshold 1 (0: <1, 1: ≥1). The NLR was treated as binary variable with threshold 3 (0: <3, 1: ≥3). In both cases, the zero category is represented by blue color and category one by red color. The median survival times (dashed lines) and the log-rank test *p*-values are also displayed in each plot.

Kaplan–Meier curves for PFS and OS, according to the KELIM score and NLR values, are shown in Figure 2. Specifically, patients with a favorable KELIM score (≥1) exhibited statistically significantly better PFS compared to those with an unfavorable score (<1) (Figure 2A), with median survival times of 41.13 months and 17.18 months, respectively (log-rank test *p*-value = 0.012). In contrast, no statistically significant difference in PFS was observed based on NLR values, with patients with NLR ≥ 3 exhibiting worse PFS compared to those with NLR < 3 (Figure 2B), with median survival times of 27.99 months and 35.22 months, respectively (log-rank test *p*-value = 0.530). When assessing OS, patients with a favorable KELIM score had statistically significantly better outcomes compared to those with an unfavorable score (Figure 2C), with median OS times of not-yet-reached and 48.30 months, respectively (log-rank test *p*-value = 0.039). However, no statistically significant difference in OS was observed between patients with NLR ≥ 3 and those with NLR < 3 (Figure 2D), with median OS times of 63.74 months and not-yet-reached, respectively (log-rank test *p*-value = 0.760).

In addition, univariable Cox regression analysis was performed for KELIM score and NLR values regarding PFS and OS, and the results are displayed in Table 2. The analysis showed that the estimated hazard ratio (HR) was statistically significantly lower for patients with a KELIM score ≥1 compared to those with a KELIM score <1 (reference category) in both recurrence and OS. Specifically, the HR values were 0.480 (95% CI: 0.266–0.864, *p* = 0.015) for recurrence and 0.453 (95% CI: 0.209–0.981, *p* = 0.045) for OS (Table 2). In other words, patients with a favorable KELIM score had less than half the hazard for both recurrence and death compared to those with an unfavorable KELIM score. By assessing the prognostic value of the variables age, BMI, CCI, ICU admission, Clavien–Dindo classification, and hospital stay, regarding both PFS and OS using univariable Cox regression, it was observed that neither of these variables exhibited a statistically significant effect on either OS or PFS. Therefore, we did not proceed in performing multivariable Cox regression analysis including any of these variables along with KELIM, since they failed to exhibit statistical significance at a univariable level.

On the other hand, patients with an NLR ≥ 3 did not show a statistically significant difference in HR for recurrence or overall survival compared to those with an NLR < 3 (reference category). More specifically, the estimated HR was 1.218 (95% CI: 0.660–2.245, *p* = 0.528) for recurrence and 1.132 (95% CI: 0.504–2.541, *p* = 0.764) for overall survival. Although, the HR was greater than 1 in both cases, indicating a potential tendency toward a higher risk for both recurrence and death for patients with an NLR ≥ 3; this result was not supported by statistical significance. Next, to evaluate whether the common threshold of three was an appropriate choice for NLR based on our data, ROC analysis was performed. It was found that in the case of recurrence, the area under the ROC (AUROC) curve was 0.507 (*p* = 0.915), while for overall survival, the AUROC curve was found to be 0.527 (*p* = 0.695). This indicated that the NLR was not able to successfully discriminate the existence or not of either recurrence or overall survival. Additionally, based on the maximally selected rank statistics procedure, regarding the recurrence, the estimated threshold for NLR was found to be 3.75 (*p* = 0.972). Regarding the overall survival, the estimated threshold was found to be 3.34 (*p* = 0.814). This shows that, although in the proximity of three, the estimated thresholds for NLR were not statistically validated to exhibit a discriminating effect regarding either PFS or OS.

Consequently, the prognostic value of the KELIM score was much more evident than that of the NLR, when considering both PFS and OS as endpoints.

## 4. Discussion

In this study, we investigated the prognostic value of KELIM score and NLR in survival outcomes for 78 patients with high-grade serous AOC treated with platinum-based NACT followed by IDS. The results showed that KELIM score ≥1 was statistically significantly associated with improved PFS and OS in these patients compared to those with a KELIM score <1. Namely, patients with a favorable KELIM score (≥1) had a reduced risk of both recurrence and death compared to those with an unfavorable score (<1). In contrast, no statistically significant difference was observed in PFS and OS among the patients based on NLR values. Although no statistically significant association was found between NLR values and the risk of recurrence or death, patients with NLR ≥ 3 exhibited a greater likelihood of recurrence and death compared to those with NLR < 3.

The KELIM score has been widely used in several studies as a prognostic marker in ovarian cancer, serving as a predictor of chemosensitivity after primary debulking [10,29] or as a predictor of complete cytoreduction in the setting of IDS after NACT [30]. In addition, its prognostic value is further supported by its impact on survival outcomes. In the present study, the association between PFS, OS, and KELIM score was analyzed, revealing a statistically significant improvement in both PFS and OS among patients with a favorable KELIM score compared to those with an unfavorable score. These findings are consistent with previous research demonstrating that a higher KELIM score (≥1) is associated with improved PFS and OS in patients undergoing IDS after NACT [10,31,32]. Additionally, a meta-analysis involving 5884 patients validated the KELIM score as a strong predictor of patient survival, regardless of the completeness of surgery. A favorable KELIM score (≥1.0) was significantly associated with longer OS (validation set: median 78.8 vs. 28.4 months; hazard ratio [HR] 0.46; 95% confidence interval [CI], 0.41–0.50; C-index 0.68) and longer PFS (validation set: median 30.5 vs. 9.8 months; HR 0.49; 95% CI, 0.45–0.54; C-index 0.68). These outcomes are in agreement with those reported in the present analysis [9].

Increasing evidence suggests that systemic inflammation and immune cells play a crucial role in cancer progression and can serve as prognostic indicators for malignancies [15]. Several components of a CBC, such as the NLR, have been explored in predicting cancer outcomes. Neutrophilia has been associated with protumoral effects, like invasion, proliferation, and metastasis. In contrast, lymphocytes play a crucial role in tumor defense and inhibition of tumor proliferation and migration [33]. Kim et al. [27] were the first to assess the prognostic value of pretreatment NLR in patients with AOC undergoing NACT. Their study demonstrated that an elevated NLR (>3.81) was associated with poor OS but not PFS. However, an association was observed between the dynamic change of the NLR during NACT and PFS. Several studies have confirmed these findings [28,34], although different NLR cut-off values were used in each study. On the contrary, a meta-analysis with 2892 patients showed that a high pretreatment NLR was statistically significantly associated with shorter PFS and OS [35].

The present study, which included patients that underwent IDS after receiving three or four cycles of NACT, exhibited that elevated NLR values were related to increased risk of recurrence or death. However, it failed to demonstrate a statistically significant association between elevated NLR and survival outcomes. OS and PFS in these patients were explored based on NLR values, using a cut-off value of 3, as established in previous studies [18,20,25,26,27,28]. Targeted analyses (ROC and maximally selected rank statistics) were conducted as well to assess the usage of alternative cut-off values for NLR, without leading into an appropriate choice. These finding suggest that NLR did not serve as a reliable predictor for survival outcomes in this specific patient cohort. The study′s sample size and design limitations might have, however, affected the statistical power of the analyses to detect this effect. Additionally, unaccounted inflammatory confounding factors—such as underlying infections, autoimmune conditions, or other systemic inflammatory processes—may have played a pivotal role in modulating NLR’s predictive capacity, further complicating its interpretation in this clinical context.

Although the primary aim of this study was to assess the prognostic value of the KELIM score and the NLR values regarding both PFS and OS, the prognostic value of the variables age, BMI, CCI, ICU admission, Clavien–Dindo classification, and hospital stay was evaluated as well to investigate the possibility of using multivariable Cox regression to account for potential confounders. Neither of these variables demonstrated a significant effect on either OS or PFS; therefore, we did not proceed in performing multivariable Cox regression analysis including any of these variables along with KELIM.

The strength of this study is that it included patients with AOC who underwent debulking surgery at a certified gynecological oncology center, accredited for advanced ovarian cancer surgery. The surgeries were performed by two proficient gynecological oncology surgeons. Furthermore, the chemotherapy treatments were administered at a certified medical oncology center, under the supervision of a professor of medical oncology, in accordance with the latest clinical guidelines. Patient follow-up was conducted as part of routine monitoring within the same hospital complex by the treating physicians. In addition, both the measurement of CA-125 levels for calculating the KELIM score and the analysis of CBC for NLR calculation were conducted in the hospital′s central laboratory, ensuring consistency in these measurements.

## 5. Conclusions

The KELIM score could be used as a predictive tool for survival outcomes in patients with AOC undergoing NACT. In our cohort, KELIM demonstrated significant and better prognostic value than NLR. Elevated NLR was not predictive of survival outcomes in contrast to findings from previous studies. Given the sample size of our cohort, large-scale studies in patients with AOC are essential to further validate these findings and determine their clinical relevance.

### Limitations of the Study

Certain limitations of our study should be considered. The retrospective design may have introduced selection bias and confounding factors. In addition, the sample size of the study (n = 78) may have limited the statistical power of the conducted analyses to detect the effects under investigation.

## Figures and Tables

**Table 1:** Patient characteristics. For quantitative variables, the mean is displayed with standard deviation in parenthesis. For qualitative variables, the frequency is displayed per category with the corresponding percentage in parenthesis. For some variables, the median and the interquartile range (IQR) are displayed as well.

Characteristics	Measures
Age	61.45 (12.32)
BMI	28.25 (5.92)
CCI	median: 2 (IQR: 1–4)
0–2	40 (51.3%)
3–4	24 (30.8%)
≥5	14 (17.9%)
ICU Admission	
No	64 (82.1%)
Yes	14 (17.9%)
Clavien–Dindo Classification	22.87 (16.24) median: 24.20 (IQR: 12.20–32.00)
Hospital Stay	8.54 (4.07) median: 8.00 (IQR: 6.00–9.00)
PFS (in months)	
N of events	45
median estimate	29.86
OS (in months)	
N of events	26
median estimate	63.74

**Table 2:** Univariable Cox regression analysis for (A) recurrence with KELIM score ≥1, (B) recurrence with NLR ≥ 3, (C) overall survival with KELIM ≥ 1, and (D) overall survival with NLR ≥ 3. The hazard ratio (HR) along with the corresponding 95% confidence interval (CI) and *p*-value are displayed.

			HR	*p*-Value	95% CI for HR	
					Lower	Upper
(A)	recurrence	KELIM ≥1	0.480	0.015	0.266	0.864
(B)	recurrence	NLR ≥3	1.218	0.528	0.660	2.245
(C)	OS	KELIM ≥1	0.453	0.045	0.209	0.981
(D)	OS	NLR ≥ 3	1.132	0.764	0.504	2.541

In all analyses, the reference category was either KELIM < 1 or NLR < 3.

## Data Availability

We confirm that the data supporting and the findings of this study are available upon request. However, due to privacy concerns and in accordance with ethical standards and regulations, the data will be provided in a deidentified format to ensure patient anonymity. Requests for data should be directed to the corresponding author via email at “theodoulidisvasilis@yahoo.gr”.

## Abbreviations

The following abbreviations are used in this manuscript:

AOC	Advanced ovarian cancer
OS	Overall survival
PDS	Primary debulking surgery
NACT	Neoadjuvant Chemotherapy
IDS	Interval debulking surgery
PFS	Progression-free survival
NLR	Neutrophil–lymphocyte ratio
KELIM	ELIMination rate constant K
CBC	Complete blood counts
ESGO	European Society of Gynecological Oncology
BMI	Body mass index
CCI	Charlson comorbidity index
ICU	Intensive care unit
PCI	Peritoneal cancer index
HR	Hazard ratio
CI	Confidence interval
IQR	Interquartile range
CA-125	Cancer antigen-125
